# Blood pressure and endothelial function in healthy, pregnant women after acute and daily consumption of flavanol-rich chocolate: a pilot, randomized controlled trial

**DOI:** 10.1186/1475-2891-12-41

**Published:** 2013-04-08

**Authors:** Jaime Andres Mogollon, Emmanuel Bujold, Simone Lemieux, Mélodie Bourdages, Claudine Blanchet, Laurent Bazinet, Charles Couillard, Martin Noël, Sylvie Dodin

**Affiliations:** 1 St. François d’Assise Hospital, Centre hospitalier universitaire de Québec (CHUQ), Quebec, Canada; 2 Reproductive Biology Research Center, Research Center, Centre hospitalier de l'Université Laval (CHUL)-CHUQ, Quebec, Canada; 3 Department of Food Sciences and Nutrition, Institute of Nutraceuticals and Functional Foods (INAF), Université Laval, Quebec, Canada; 4 Public Health Research Center, CHUQ, Quebec, Canada; 5 Institute of Nutraceuticals and Functional Foods (INAF), Université Laval, Quebec, Canada; 6 Department of Obstetrics and Gynecology, Research Center, St. François d’Assise Hospital (CHUQ), Université Laval, Québec, QC, G1L 3 L5, Canada

**Keywords:** Cacao, Chocolate, Theobromine, Flavanols, Pregnant women, Hypertension, Pregnancy-induced, Preeclampsia, Epicatechin

## Abstract

**Background:**

Several randomized clinical trials (RCTs) indicate that flavanol-rich chocolate has beneficial effects on flow-mediated dilation (FMD) and blood pressure (BP). However, no RCTs have evaluated these outcomes in pregnant women. The objective of this 2-group, parallel, double-blind RCT was to examine the effects of flavanol-rich chocolate on FMD and BP in pregnant women with normal BP.

**Methods:**

Forty-four healthy, pregnant women were randomized to the high-flavanol (n = 23) or low-flavanol (n = 21) chocolate consumption for 12 weeks. At randomization (0, 60, 120 and 180 min after a single 40-g dose of chocolate), 6 and 12 weeks after daily 20-g chocolate intake, we evaluated plasma concentrations of flavanols and theobromine, as well as the FMD and BP.

**Results:**

Plasma epicatechin was significantly increased (p < 0.001) 180 min after the consumption of 40-g high-flavanol chocolate compared to low-flavanol chocolate. Theobromine concentrations were significantly higher 180 min and 12 weeks after the intake of experimental chocolate or low-flavanol chocolate (p < 0.001). FMD was not different between the 2 groups at all pre-defined time periods. No other significant within-group or between-group changes were observed.

**Conclusion:**

These results confirm the feasibility of a large-scale RCT comparing daily consumption of flavanol-rich chocolate to an equivalent placebo during pregnancy and demonstrate higher plasma epicatechin and theobromine concentration in the intervention group after acute ingestion

**Trial registration:**

ClinicalTrials.gov Identifier: NCT01659060

## Introduction

Preeclampsia (PE), defined as gestational hypertension associated with proteinuria, is one of the most common medical disorders affecting pregnancy [1], with potentially severe consequences for mother and child, especially in developing countries [2]. It is estimated that 3 to 8% of all pregnancies are impacted by this complication. Every year, PE is responsible for about 60,000 deaths worldwide [1]. More than half of women with PE will undergo caesarean delivery [1]. PE heightens the risk of peri-natal mortality by 5-fold and is a major cause of low birth weight in infants [1]. Numerous studies have suggested that women who develop PE have an increased risk of cardiovascular disease later in life [3]. The cardiovascular manifestations of PE share many characteristics and risk factors with cardiovascular disease, including hypertension, endothelium dysfunction and oxidative stress [4,5].

Despite intensive research, PE remains an idiopathic disease for which few effective prophylactic measures are available to patients [2]. PE is associated with a placental disease in most cases and is distinguished by generalized maternal dysfunction of the endothelium. Endothelium dysfunction leads to clinical symptoms in the mother [6,7]. There is strong evidence that maternal nitric oxide (NO) deficiency plays a key role in the development of PE [8]. Therapeutic approaches focusing on up-regulating NO availability may be useful targets in PE prevention. Flavanols, the most common flavonoids in dark chocolate, are potent antioxidants capable of inducing NO-dependent vasodilation. A recent meta-analysis of randomized controlled trials (RTCs) confirmed that flavanol-rich chocolate has a beneficial influence on endothelial function and reduces systolic (SBP) and diastolic blood pressure (DBP) [9]. Recent cohort [10] and case–control studies [11] have indicated that chocolate consumption during pregnancy may lower the risk of PE, but their design did not permit the exploration of temporal relationships. Moreover, this association was not found in another case–control study [12]. Finally, a non-placebo, controlled, non-blinded study by Di Renzo et al [13] suggested that modest daily intake of high-cocoa content chocolate contributes to BP reduction during pregnancy.

As endothelial dysfunction is fundamental to PE development we believe that the growing body of literature supporting the hypothesis of a beneficial outcome of flavanol-rich chocolate consumption, through its action on endothelial function and BP regulation, justifies a clinical trial in pregnant women.

The primary objective of this pilot RCT is to test the feasibility of design methods and procedures for later use on a larger scale. The secondary objective is to examine the acute and chronic impact of dark chocolate on FMD and BP in healthy, pregnant women with normal BP.

## Patients and methods

### Ethics statement

Protocol and consent form of this study was reviewed and approved by the institutional Ethics Committee of Centre Hospitalier Universitaire de Québec. An information and consent form, approved by the institutional Ethics Committee, was read and signed by the participants.

### Study participants

Between July 2008 and April 2009, we enrolled non-smoking, normotensive women aged 18 to 35 years, with a live fetus between the 7^th^ and 12^th^ weeks of gestation documented by ultrasound. Normal BP was defined as SBP <140 mmHg and DBP <90 mmHg.

We excluded patients with a family history of premature cardiovascular disease, chronic hypertension, renal dysfunction, medication use for treating hypertension or interfering with the metabolism of glucose or lipids, taking supplements or natural health products that may interfere with BP (fish oils, coenzyme Q10, and garlic). Women consuming 1 alcoholic drink per day or more, or suffering from allergies or intolerance to nuts or chocolate were also excluded.

### Recruitment and randomization

Women were recruited through advertisements given to them by healthcare professionals previously informed about the project. Those wishing to participate contacted the Study Coordinator who explained the research project to them. They then presented themselves at the Research Center of Saint-François d’Assise Hospital for a total of 4 visits.

The recruitment protocol included an initial visit during which suitability for randomization was evaluated. Inclusion and exclusion criteria as well as the risks and benefits of the study were reviewed in detail during this pre-randomization visit. An information and consent form, approved by the institutional Ethics Committee, was read and signed. BP was measured 3 times at 3-min intervals and averaged, according to a validated protocol [14]. Anthropometric measurements and fasting blood samples were collected. The blood samples were analyzed for lipid and fasting blood sugar profiles. A questionnaire documenting social and demographic characteristics, tobacco use, consumption of alcohol and medications was completed by all women which were also required to complete a 3-day food record [15], including a weekend day, and a validated 2-day activity record [16,17] the week before the 2^nd^ visit. Nutrient and food intake results were evaluated with the Nutrition Data System for Research, version 4.03, developed by the Nutrition Coordinating Center, University of Minnesota in Minneapolis.

Eligible healthy, pregnant women were randomly assigned to either high-flavanol dark chocolate (experimental group) or low-flavanol dark chocolate (placebo group). Concealed randomization was generated using computer-aided block randomization (block size was kept secret), with pre-stratification by parity and body mass index (BMI), under the responsibility of an independent statistician. Another statistician undertook treatment allocation independently of the trial team. At the randomization visit participants were instructed to consume in the fasting state 40-g of chocolate within 10 min. The composition of each chocolate bar – energy, nutrients, catechins, epicatechins and theobromine – was quantified by Barry Callebaut (Lebbeke-Wieze, Belgium) and re-evaluated before the beginning of the study at the INAF Laboratory using mass spectrometry (Table 1). All chocolate bars were standardized for their flavanol and theobromine content and matched for caloric load, nutrients and caffeine. All of them were similar in taste and were supplied free in individual, opaque packaging by Barry Callebaut. Our cocoa provided 400 mg of total flavanols. Therefore, to isolate the effects of flavanols, our chocolate placebo was identical to the experimental chocolate in its content for all other nutrients except for flavanols (including theobromine and caffeine contents).

**Table 1:** Chocolate composition (20 g)

**Components**	**High-flavanol**	**Low-flavanol**
	**chocolate**	**chocolate**
**Energy (kcal)**	102	102
**Total fat (g)**	7.5	7.5
**Carbohydrates (g)**	6.5	6.5
**Protein (g)**	1.35	1.35
**Total flavanols (mg)**	400	<60
**Total catechin + epicatechin (mg)**	64	14
**Caffeine (mg)**	23.6	23.6
**Theobromine (mg)**	150	150

### Clinical follow-up

During the chronic phase, the women were advised to consume chocolate bars 3 times a day for a total of 20-g daily providing 400 mg of total flavanols and 64 mg of epicatechin and catechin. This flavanol daily intake should be sufficient to exert beneficial effects. Indeed, in a similar study of non-pregnant women, a chronic consumption of 373 mg flavanol content was associated with significant outcomes on BP and endothelial function. Moreover, its caloric value was easily replaced by a snack of equivalent energy. Therefore, the balance between beneficial effects, caloric value and fat content was carefully considered.

Participants were scheduled for 2 follow-up visits at 6 and 12 weeks after randomization. All clinical investigations, laboratory analyses, data collection and assessment were blinded to the randomization allocation.

The participants were asked not to consume other chocolate products during the study and to avoid foods rich in polyphenols or theobromine (tea, coffee, fruit juice, wine) for 24 h before each visit. Intense physical activity was also forbidden for 48 h preceding each visit. Anthropometric measurements were taken at each visit, and the participants completed a food frequency questionnaire (FFQ) specifically evaluating their polyphenol consumption in the last month. Blood samples were collected, and FMD and BP were quantified.

## Endpoints

### FMD

The primary endpoint was change in endothelial function, measured as FMD of the brachial artery, as described previously [18,19]. A compact ultrasound system – LOGIQe from GE Healthcare Technology, and Brachial Analyser version 5 software – recorded these measurements. Accuracy and reproducibility were measured previously by other groups [20] and the coefficients of variation were 9.8%, 10.6%, 6.6%, and 9.2% at 4–6 hours, 1 week, 1 month, and 3 months, respectively. The test was performed by the same experienced technician in a quiet, dimly-lit room, between 08:00 and 12:00 h (noon). Ambient temperature was comfortable and constant (23.5°C). Before each measurement, the women lay on their back, in a comfortable position, with the arms and feet uncrossed for 15 min. They were encouraged to avoid speaking. The right arm was extended laterally for measurement at a 90-degree angle to the body. The forearm was slightly supine in a comfortable position. Depth was programmed and parameters were adjusted to obtain an optimal view of the anterior and posterior walls of the brachial artery. The ultrasound probe was positioned precisely.

Once these parameters were optimized and the probe position noted, they were recorded and kept constant throughout the study. The right brachial artery was imaged longitudinally just above the antebrachial fossa, and images were captured at the same moment in the cardiac cycle (R wave peak).

### BP

BP was measured by a trained, certified nurse blinded to treatment allocation, with an electronic monitor (Microlife 3 BTO-A) after 15 min of rest, back supported, arm supported at the heart level, and cuff placed on the left upper arm. The device achieved British Hypertension Society (BHS) A/A grade accuracy and was validated in a pregnant population, including PE, according to the BHS protocol [14]. BP was measured 3 times at 3-min intervals at the randomization visit and 0, 60, 120 and 180 min after chocolate bar intake. It was assessed with the same standardized protocol at weeks 6 and 12.

### Plasma biomarkers of chocolate intake

Methylxanthines (caffeine, theobromine and theophylline) were simultaneously quantified by high-pressure liquid chromatography (HPLC) [21], by a technician blinded to participant status. Inter- and intra-assay coefficients of variation were 5.2% and 3.7%, respectively.

Plasma flavanol concentrations were measured with standard analysis equipment. Flavanols were purified by solid extraction, followed by HPLC with a fluorescence detection system [22].

### Side-effects

Weight change was evaluated at each visit, and digestive and other signs and symptoms (nausea, abdominal pain, constipation, and headache) were documented with a self-administered questionnaire related to the week preceding the visit.

### Compliance

Each participant received a telephone reminder in the week preceding each visit. Women were paid an allocation for expenses related to the assessment visits. If a participant missed a visit, the nurse scheduled a new appointment. In addition, study participants recorded their daily intake of chocolate bars on diary cards.

### Blinding and contamination bias

The proportion of women who guessed right about group allocation was documented with a short questionnaire at the last visit. To control for contamination bias, the flavonoid consumption was measured by FFQ in the last month preceding each follow-up visit.

### Sample size and statistical analysis

Changes in FMD were defined as a primary endpoint calculated as variations 12 weeks after chocolate intake and expressed as mean differences. The secondary outcome was BP. All analyses were based on intention-to-treat. In the context of this feasibility study, no sample size was calculated and the 44 pregnant women who agreed to participate were included on the basis of recommendations by Thabane et al [23].

The baseline characteristics of the 2 groups were compared, and factors influencing BP were considered descriptively. Non-paired *t* tests were performed for planned comparisons between groups. Changes within groups were evaluated by paired *t* test. P ≤ 0.05 values were considered as significant. The presence of side-effects was compared in the 2 groups.

## Results

**Figure 1:**
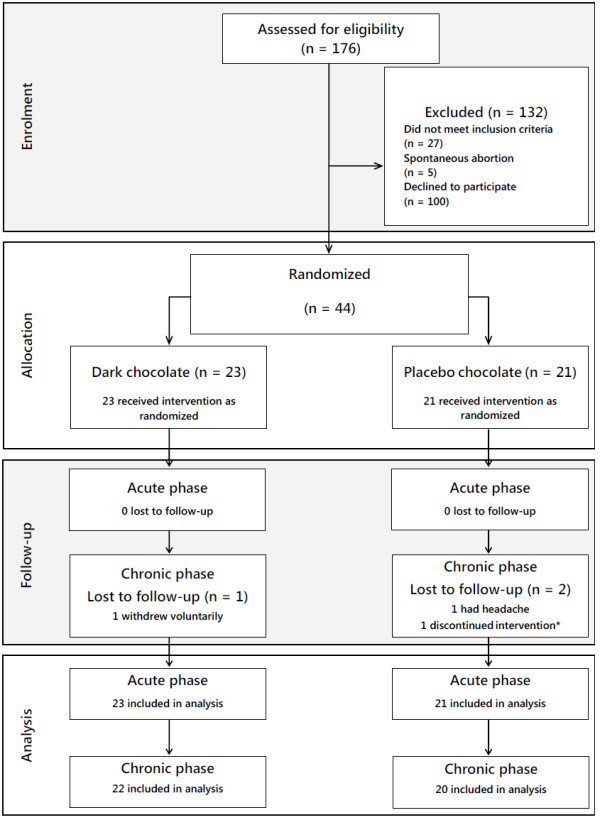
**Flow diagram of study participants.** *The participant withdrew voluntarily from the study without giving a reason, but accepted to return.

We approached 176 women, of whom 27 were excluded as they failed to meet the inclusion criteria: 12 were over 16 weeks pregnant, 6 were smokers, 5 had a body mass index >30, 4 did not meet other criteria, and 5 had spontaneous abortions (n = 5). One hundred women declined to participate for different reasons: 72 women had logistical or personal problems (transportation, work, other children at home, too time-consuming), 18 did not return calls, and 10 refused to give a reason for withdrawing their participation. Finally, 44 (33%) of the 132 pregnant women who met the inclusion criteria and who agreed to participate were randomised (23 in the high-flavanol chocolate arm and 21 in the low-flavanol chocolate arm) (Figure 1). The baseline characteristics of the women who declined to participate were not significantly different from those of randomised women (data not included). At randomization, the 2 arms were well balanced with regard to social and baseline characteristics (Tables 2 and 3).

**Table 2:** Demographic characteristics

**Characteristics**	**High-flavanol chocolate (n = 23)**	**Low-flavanol chocolate (n = 21)**
**Marital status**		
Common-law spouse	19 (82.6)	15 (71.4)
Married	4 (17.4)	6 (28.6)
**Number of children**		
0	14 (60.9)	13 (61.9)
1	7 (30.4)	6 (28.6)
2	2 (8.7)	1 (4.8)
3	0	1 (4.8)
**Educational attainment**		
University degree	17 (73.9)	15 (71.4)
College or trade certification	5 (21.7)	4 (19.1)
Some university education	0	1 (4.8)
Some college education	0	1 (4.8)
High school diploma	1 (4.4)	0
Women with paid work^*^	23 (100)	19 (90.5)
**Average hours worked (per year)**^**˦**^	1,717.1 ± 482.7	1,817.5 ± 503.9
	(n = 22)	(n = 19)
**Perception of financial situation **^**˦˦**^		
Sufficient income	13 (56.5)	13 (61.9)
Low income	10 (43.5)	8 (38.1)
**Regular alcohol consumption **^**˦˦˦**^	4 (17.4)	1 (4.8)
**Smoker**^**˦˦˦**^	0	1 (4.8)

^*^ During the past year Number (percentage).

^˦^ Values are means ± SD.

^˦˦^ Perception of women compared to people of their age.

^˦˦˦^ In the last month.

**Table 3:** Baseline characteristics of 44 pregnant women by study arm

**Baseline characteristics**	**High-flavanol chocolate (n = 23)**	**Low-flavanol chocolate (n = 21)**
Age (years)	28.7 ± 3.17	29.76 ± 3.63
Height (m)	1.64 ± 0.06	1.66 ± 0.04
Weight (kg)	66.07 ± 6.79	64.90 ± 8.14
BMI (kg/m^2^)	24.73 ± 2.68	23.61 ± 2.85
Gestation duration at randomisation (weeks)	21.13 ± 1.10	21.10 ± 1.61
**Blood pressure**		
Systolic blood pressure (mmHg)^*^	109.43 ± 7.65	105.94 ± 7.84
Diastolic blood pressure (mmHg)^*^	66.80 ± 6.34	64.23 ± 6.24
Mean arterial pressure (mmHg)^*^	81.01 ± 5.87	78.14 ± 6.48
**Flow-mediated dilation** (%)	11.86 ± 4.66	13.25 ± 3.80
**Plasma biomarkers of chocolate intake**		
Theobromine (μg/mL plasma)	0.45 ± 0.43	0.35 ± 0.37
Theophylline (μg/mL plasma)	0.13 ± 0.18	0.07 ± 0.11
Caffeine (μg/mL plasma)	0.58 ± 0.82	0.29 ± 0.46
**Plasma flavanol concentrations**		
Epicatechin (ng/mL plasma)	4.90 ± 0.00	4.90 ± 0.00
Catechin (ng/mL plasma)	55.99 ± 44.23	44.05 ± 46.68

^*^Average of 3 measurements Values are means ± SD.

Of the 44 randomized women who participated in the acute phase, 42 (22 and 20 in the high-flavanol and low-flavanol chocolate arms, respectively) had outcomes available for intention-to-treat analysis. Two women dropped out of the study for reasons not related to the intervention.

### Plasma biomarkers of chocolate intake

Plasma epicatechin concentrations increased significantly in the high-flavanol chocolate group at 180 min after intervention compared to low-flavanol chocolate (p < 0.001) (Table 4). This dose of high-flavanol chocolate in the current study had no effect on increasing plasma catechin concentrations. After 12 weeks, epicatechin and catechin concentrations were too low to be detectable.

**Table 4:** Acute changes in the plasma concentrations of biomarkers after single dose chocolate intake

	**Low-flavanol**	**High-flavanol**	***P *value of change between treatments**
	**Chocolate**	**Chocolate**	
	**(n = 21)**	**(n = 23)**	
**Epicatechins** (ng/mL)			
0 min (Baseline)	4.90 ± 0.00	4.90 ± 0.00	-
180 min	4.90 ± 0.00	97.02 ± 41.94	<0.0001
*P* value (180 *vs.* 0 min)	-	<0.0001	
**Catechins** (ng/mL)			
0 min (Baseline)	44.05 ± 46.68	55.99 ± 44.23	**-**
180 min	43.62 ± 42.88	59.11 ± 43.50	0.70
*P* value (180 *vs.* 0 min)	0.92	0.70	

Values are means ± SD.

As expected, methylxanthine concentrations were significantly higher at 180 min compared to baseline in the 2 groups (Table 5). More specifically, a significant increase in plasma theobromine concentrations was observed in both groups at 180 min and was slightly but significantly more marked in the experimental group. Therefore, theobromine concentrations served as a marker of chocolate compliance. A significant difference in caffeine concentrations (p = 0.02) was also seen in the two chocolate groups after single chocolate intake.

**Table 5:** Acute changes in methylxanthine concentrations after single-dose chocolate intake

	**Low-flavanol**	**High-flavanol**	***P *value of change between treatments**
	**chocolate**	**chocolate**	
	**(n = 21)**	**(n = 23)**	
**Theobromine** (μg/mL)			
0 min (baseline)	0.35 ± 0.37	0.45 ± 0.43	-
180 min	5.47 ± 1.29	6.51 ± 0.80	0.0122
*P* value (180 *vs.* 0 min)	<0.0001	<0.0001	
**Theophylline** (μg/mL)			
0 min (Baseline)	0.07 ± 0.11	0.13 ± 0.18	**-**
180 min	0.12 ± 0.12	0.18 ± 0.13	0.85
*P* value (180 *vs.* 0 min)	<0.0001	0.0009	
**Caffeine** (μg/mL)			
0 min (Baseline)	0.29 ± 0.46	0.58 ± 0.82	**-**
180 min	1.07 ± 0.50	1.54 ± 0.67	0.0201
*P* value (180 *vs.* 0 min)	<0.0001	<0.0001	

Values are means ± SD.

Compared to baseline, theobromine concentrations were still significantly higher in high-flavanol and low-flavanol chocolate groups at 6 and 12 weeks, indicating good subject compliance (Table 6). A small but significant difference in plasma theobromine concentrations (p = 0.03) was noted between chocolate groups after 12 weeks of chocolate consumption.

**Table 6:** Chronic changes in methylxanthine concentrations after daily consumption of 20-g chocolate

	**Low-flavanol**	**High-flavanol**	***P *value of change between treatments**
	**Chocolate**	**Chocolate**	
	**(n = 20)**	**(n = 22)**	
**Theobromine** (μg/mL)			
Baseline	0.35 ± 0.37	0.45 ± 0.43	-
Week 6	1.44 ± 0.73	1.82 ± 0.76	0.34
Week 12	1.30 ± 0.69	1.87 ± 0.85	0.03
*P* value (Week 6 *vs.* Baseline)	<0.0001	<0.0001	
*P* value (Week 12 *vs.* Baseline)	<0.0001	<0.0001	
**Theophylline** (μg/mL)			
Baseline	0.07 ± 0.11	0.13 ± 0.18	**-**
Week 6	0.24 ± 0.24	0.27 ± 0.29	0.66
Week 12	0.17 ± 0.17	0.27 ± 0.20	0.49
*P* value (Week 6 *vs.* Baseline)	0.0001	0.045	
*P* value (Week 12 *vs.* Baseline)	0.0003	0.0076	
**Caffeine** (μg/mL)			
Baseline	0.29 ± 0.46	0.58 ± 0.82	**-**
Week 6	1.12 ± 1.27	1.30 ± 2.08	0.78
Week 12	0.82 ± 1.15	1.56 ± 2.05	0.34
*P* value (Week 6 *vs.* Baseline)	0.004	0.12	
*P* value (Week 12 *vs.* Baseline)	0.02	0.03	

Values are means ± SD.

### FMD and BP

FMD, SBP, DBP and mean arterial BP (MAP) were not significantly affected by acute or chronic consumption of high-flavanol chocolate, as reported in Tables 7 and 8.

**Table 7:** Acute changes in FMD and BP after single dose chocolate intake

	**Low-flavanol**	**High-flavanol**	***P *value of change between treatments**
	**Chocolate**	**Chocolate**	
	**(n = 21)**	**(n = 23)**	
**FMD (%)**			
0 min (baseline)	13.3 ± 3.8	11.9 ± 4.7	-
60 min	13.0 ± 4.3	10.2 ± 3.1	0.50
120 min	12.1 ± 3.0	10.8 ± 4.2	0.99
180 min	11.0 ± 2.8	11.6 ± 3.7	0.18
*P* value (60 *vs.* 0 min)	0.82	0.18	
*P* value (120 *vs.* 0 min)	0.35	0.24	
*P* value (180 *vs.* 0 min)	0.16	0.77	
**Systolic BP** (mmHg)			
0 min (baseline)	105.9 ± 7.8	109.4 ± 7.6	**-**
60 min	107.0 ± 5.7	110.3 ± 6.8	0.95
120 min	105.3 ± 6.0	110.0 ± 7.1	0.49
180 min	105.8 ± 5.7	108.7 ± 6.3	0.72
*P* value (60 *vs.* 0 min)	0.38	0.39	
*P* value (120 *vs.* 0 min)	0.59	0.66	
*P* value (180 *vs.* 0 min)	0.86	0.55	
**Diastolic BP** (mmHg)			
0 min (baseline)	64.2 ± 6.2	66.8 ± 6.3	**-**
60 min	64.2 ± 5.9	64.5 ± 5.1	0.07
120 min	64.0 ± 5.1	66.1 ± 5.6	0.68
180 min	64.2 ± 4.9	66.4 ± 5.1	0.81
*P* value (60 *vs.* 0 min)	0.98	0.01	
*P* value (120 *vs.* 0 min)	0.73	0.36	
*P* value (180 *vs.* 0 min)	0.94	0.68	
**Mean arterial BP** (mmHg)			
0 min (baseline)	78.1 ± 6.5	81.0 ± 5.9	**-**
60 min	78.5 ± 5.4	79.8 ± 5.2	0.16
120 min	77.8 ± 5.1	80.7 ± 5.7	0.92
180 min	78.0 ± 4.8	80.5 ± 4.9	0.73
*P* value (60 *vs.* 0 min)	0.72	0.08	
*P* value (120 *vs.* 0 min)	0.64	0.69	
*P* value (180 *vs.* 0 min)	0.90	0.52	

Values are means ± SD.

**Table 8:** Changes in FMD and BP after daily consumption of 20-g chocolate for 6 and 12 weeks

	**Low-flavanol**	**High-flavanol**	***P *value of change between treatments**
	**Chocolate**	**Chocolate**	
	**(n = 20)**	**(n = 22)**	
**FMD (%)**			
Baseline	13.3 ± 3.8	11.9 ± 4.7	-
Week 6	11.4 ± 3.4	10.8 ± 3.9	0.22
Week 12	13.1 ± 3.8	10.4 ± 3.4	0.43
*P* value (Week 6 *vs.* Baseline)	0.06	0.32	
*P* value (Week 12 *vs.* Baseline)	0.95	0.18	
**Systolic BP** (mmHg)			
Baseline	105.9 ± 7.8	109.4 ± 7.6	**-**
Week 6	104.8 ± 6.0	109.4 ± 7.5	0.98
Week 12	106.1 ± 9.7	108.8 ± 8.9	0.49
*P* value (Week 6 *vs.* Baseline)	0.69	0.83	
*P* value (Week 12 *vs.* Baseline)	0.66	0.60	
**Diastolic BP** (mmHg)			
Baseline	64.2 ± 6.2	66.8 ± 6.3	**-**
Week 6	65.4 ± 5.9	68.0 ± 5.0	0.51
Week 12	68.2 ± 6.9	70.5 ± 6.8	0.50
*P* value (Week 6 *vs.* Baseline)	0.05	0.42	
*P* value (Week 12 *vs.* Baseline)	0.0007	0.01	
**Mean arterial BP** (mmHg)			
Baseline	78.1 ± 6.5	81.0 ± 5.9	**-**
Week 6	78.5 ± 5.6	81.8 ± 5.2	0.65
Week 12	80.9 ± 7.5	83.2 ± 7.1	0.45
*P* value (Week 6 *vs.* Baseline)	0.20	0.64	
*P* value (Week 12 *vs.* Baseline)	0.0081	0.15	

Values are means ± SD.

No significant changes were observed within or between groups for SBP after 6 or 12 weeks. Although within normal values, DBP increased significantly from baseline after 6 and 12 weeks only in the low-flavanol chocolate group, but the difference between the 2 groups was not significant. At 12 weeks, MAP rose significantly in the low-flavanol chocolate group compared to baseline, but the difference between the 2 arms was not significant. No other significant BP changes were observed between or within groups.

### Side-effects

High-flavanol and low-flavanol chocolate did not significantly increase the intake of energy, proteins, carbohydrates and fats after 12 weeks of chocolate consumption (Tables 9). Total energy consumed by pregnant women was similar in both groups. A significant decrease in the percentage of calories from proteins in the low-flavanol chocolate group (difference: -1.7; 95% CI: -2.8 to −0.5) was apparent after the intervention (Table 9). The contribution of other macronutrients to total energy intake did not change over time for any of the two groups studied.

**Table 9:** Differences in energy and macronutrient intakes after 12 weeks of chocolate consumption

	**Low-flavanol**	**High-flavanol**	***P *value of change between treatments**
	**Chocolate**	**Chocolate**	
	**(n = 20)**	**(n = 22)**	
**Total energy** (Kcal)			
Baseline	2,086 ± 329	2,320 ± 464	-
Week 12	2,178 ± 286	2,251 ± 465	0.18
*P* value (Week 12 *vs.* Baseline)	0.28	0.42	
**Proteins** (g)			
Baseline	88.3 ± 19.7	89.4 ± 18.2	**-**
Week 12	82.7 ± 13.8	85.0 ± 22.2	0.86
*P* value (Week 12 *vs.* Baseline)	0.12	0.39	
**Proteins** (% energy)			
Baseline	17.0 ± 2.6	15.6 ± 2.4	**-**
Week 12	15.3 ± 2.3	15.1 ± 2.2	0.86
*P* value (Week 12 *vs.* Baseline)	0.13	0.0078	
**Carbohydrates** (g)			
Baseline	272.2 ± 45.0	305.8 ± 62.8	**-**
Week 12	284.5 ± 38.1	297.6 ± 64.0	0.19
*P* value (Week 12 *vs.* Baseline)	0.33	0.39	
**Carbohydrates** (% energy)			
Baseline	52.5 ± 5.6	53.0 ± 5.3	**-**
Week 12	52.4 ± 3.7	53.0 ± 4.9	0.10
*P* value (Week 12 *vs.* Baseline)	0.92	0.99	
Baseline	77.3 ± 17.6	89.2 ± 26.9	**-**
Week 12	83.2 ± 16.3	86.3 ± 23.1	0.25
*P* value (Week 12 *vs.* Baseline)	0.22	0.61	
**Lipids** (% energy)			
Baseline	33.1 ± 3.8	34.2 ± 5.2	**-**
Week 12	34.2 ± 3.5	34.3 ± 3.7	0.28
*P* value (Week 12 *vs.* Baseline)	0.35	0.92	

Values are means ± SD.

In both arms, body weight and body mass index (BMI) increased significantly after chocolate consumption for 12 weeks (Table 10), but no significant differences in these parameters were observed between the high-flavanol and low-flavanol chocolate arms.

**Table 10:** Body weight and BMI after chocolate consumption for 12 weeks

	**Low-flavanol**	**High-flavanol**	***P *value of change between treatments**
	**Chocolate**	**Chocolate**	
	**(n = 20)**	**(n = 22)**	
**Weight** (kg)			
Baseline	64.1 ± 7.5	66.6 ± 6.5	-
Week 12	70.2 ± 8.2	72.9 ± 7.2	0.74
*P* value (Week 12 *vs.* Baseline)	<0.0001	<0.0001	
**BMI** (kg/m^2^)			
Baseline	23.3 ± 2.6	24.9 ± 2.6	**-**
Week 12	25.5 ± 2.9	27.2 ± 2.8	0.58
*P* value (Week 12 *vs.* Baseline)	<0.0001	<0.0001	

Values are means ± SD.

## Discussion

In healthy, pregnant women with normal BP and without risk of PE, acute and chronic consumption of flavanol-rich chocolate was not associated with significant changes in either FMD, SBP or DBP. Our clinical study provides important insights into the feasibility, acceptability and methodology of a larger clinical trial to evaluate long-term chocolate intake in pregnant women. Our results confirmed the feasibility and good compliance of our intervention and its effectiveness in increasing blood theobromine and flavanol metabolite concentrations after acute ingestion.

The association between chocolate consumption and the risk of PE was explored for the first time in a cohort study [10] of 2,291 pregnant North American women who gave birth to a single, living baby. “Exposure” to chocolate was measured in 2 ways: data on the provision of caffeine-rich drinks and chocolate consumption since the beginning of pregnancy were collected during a structured interview around the 14th week of gestation. Consumption was also evaluated during the 3rd trimester of pregnancy in a post-natal interview. On the other hand, the serum concentrations of theobromine were quantified in a blood sample collected from the umbilical cord during delivery. Serum theobromine concentrations were inversely correlated with the risk of PE before and after adjustment for the main confounding factors (odds ratio (OR) = 0.31; 95% CI: 0.11 - 0.87, for the highest compared to the lowest quartile). Women who consumed 5 or more portions of chocolate per week during the 3rd trimester of pregnancy manifested a 40% decrease in PE risk compared to women who consumed less than 1 portion of chocolate per week (OR = 0.60; 95% CI: 0.30 - 1.24). However, this diminution was not significant. A case–control study [12] nested in a cohort does not support the previous finding. According to the authors, unmeasured confounding or reverse causation may account for the positive association previously reported as discussed above. A non-placebo, controlled, non-blinded study by Di Renzo et al [13] suggested that modest daily intake of high-cocoa content chocolate contributes to BP reduction during pregnancy. The results of these epidemiologic studies reinforce the importance to assess the effect of chocolate consumption using a clinical trial design.

In our study, DBP increased significantly from baseline to 12 weeks in the high-flavanol and low-flavanol chocolate groups, but the difference between the 2 groups was not significant. It is probable that this elevation was related to the normal increases from the second to the third trimester of pregnancy.

Although no prospective studies in pregnant women are available, the effects of daily flavanol-rich chocolate intake on endothelial function and BP have already been well documented in other populations [24,25]. A more recent meta-analysis by Hooper et al., that included 11 studies and 373 participants, suggested improvement of FMD 2 h after chocolate ingestion and its chronic intake [26]. Stronger effects were apparent at higher doses of epicatechin that could be a key contributor to the outcomes observed. They also found a significant effect of chocolate on DBP and MAP but heterogeneity of the results of the 11 studies included in this meta-analysis was significant. This heterogeneity could be partially explained by BP differences in participants at the start of chocolate intake. Indeed, recent investigations have reported unchanged [27] or no significant change [28] in SBP and DBP after dark chocolate consumption in normotensive, healthy populations. Taubert et al., in their meta-analysis, observed trends towards a marked effect of chocolate intake in patients with high BP [29].

In our clinical trial, although acute ingestion of our experimental chocolate bars was accompanied by a very significant increase in plasma epicatechin concentrations, daily, chronic epicatechin intake by pregnant women could have been too low to improve BP. In fact, in the meta-analysis by Hooper et al [26], subgroup analysis by epicatechin dose suggested greater effects on BP at doses exceeding 50 mg, but not with doses less than 50 mg. In our study, pregnant women had to consume 20-g of chocolate daily, which provided less than 50 mg of epicatechin if all chocolate bars provided were eaten.

Other investigations also showed an increment in plasma epicatechin concentrations after acute consumption of flavanol-rich products [30-32]. The amount of experimental chocolate administered in our study had no effect on increasing plasma catechin concentrations. Similar observations have been made previously [30,31]. Epicatechin is the predominant flavanol in dark chocolate. Moreover, among the flavanols, epicatechin has been reported to occur in a primary bioavailable form [33]. Holt et al [32] suggested that complex mixtures of chocolate dimers and oligomers may be degraded in the gut into epicatechin monomers, which promotes absorption and would explain the low catechin concentrations in plasma.

At 12 weeks, epicatechin was below the limit of detection. Blood sample extraction after a 12-h fast could have been responsible for the low epicatechin concentrations, considering that epicatechin clearance from the plasma compartment is very fast [31] and most absorbed epicatechin is cleared from the blood by that time [30,34]. Moreover, Taubert et al [35] excluded the storage of plasma phenols after dark chocolate intake because the short elimination half-lives of flavanols prevented accumulation of plasma levels and thus no flavanols could be detected after 12-h post-intake of flavonoid-rich dark chocolate. Nevertheless, after daily dietary chocolate incorporation for 12 weeks, even if epicatechin concentrations were very low, plasma theobromine increased 4-fold, indicating very good compliance, as discussed below. Plasma epicatechin levels should be quantified 2 h after the chocolate dose because of the short elimination half-life of flavanols.

The marginal but statistically-significant increase in theobromine levels in the experimental compared to placebo group at 180 minutes and 12 weeks post-randomisation could be attributed to different rates of intestinal metabolism, which is influenced by diet. Indeed, orally-administered, theobromine is rapidly and almost completely absorbed by the gastrointestinal tract [31]. At baseline, theobromine concentrations were also slightly lower in the placebo group, but its sources in diet measured by FFQ in the last month preceding randomisation and each follow-up visit, were not significantly different between the 2 groups.

Some limitations [36] of the technique for FMD assessment could also explain our lack of significant effect. Measurement of endothelial function is challenging. Although a number of non-invasive techniques are now available, the most frequently-used method involving FMD of the brachial artery with ultrasound imaging was incorporated in our trial. This gold standard technique is not easy and requires extensive sonographer training as well as labour-intensive image analysis. Also, lack of standardization could influence methodological reproducibility in follow-up studies, particularly among pregnant women. Moreover, in this trial, we adopted M mode during image acquisition of the brachial artery. However, both M and A modes are applied to continuously measure brachial diameter, and these techniques may be subject to error due to tracking drift [36]. In addition, M mode is more variable than B mode; thus, it is difficult to maintain the positional stability of measurements which must be performed at set time points (50 s), limiting reproducibility and showing lower FMD as a result [20]. Furthermore, 40 to 60 patients are typically needed in a parallel-group study design to find significant improvement in FMD [36]. Sample size in our study was likely too small to detect a significant difference between the 2 groups with this method. More novel fingertip-based methodologies, such as pulse amplitude tonometry, which has the potential advantage of an automated, computerized analysis system that minimizes operator dependency and inter-observer variability, would be more appropriate in prospective studies.

It is relevant to point out and discuss the consumption of low-flavanol chocolate bars as placebo to high-flavanol chocolate bars. In fact, our placebo chocolate probably did not have a neutral effect compared to experimental chocolate because it provided similar theobromine concentrations [26]. Although the beneficial outcome of chocolate on FMD and BP has been largely associated with flavanols, a recent clinical trial indicated that theobromine could be partly responsible for the BP-lowering action of chocolate [37]. Our results could have been masked by its effects, which would confirm the importance of carefully selecting an adequate placebo.

Interestingly, after checking the medical records of participants when the study ended, the pregnant women high-flavanol chocolate group had not incurred any kind of disease related to hypertension or PE, whereas a participant in the low-flavanol chocolate group had PE during late pregnancy (result not shown).

Important and significant theobromine concentrations, a good marker of chocolate intake, and the count of unused chocolate bars confirmed good compliance with the intervention during 12-week follow-up, which did not differ across arms. This finding suggests that 20-g chocolate bars could be incorporated in the usual diet of pregnant women for as long as 12 weeks. Based on the compliance difference reported by pregnant women (97.6% vs. 93.6%, experimental and placebo group, respectively) the slight difference at week 12 (p = 0.03) between the concentrations of theobromine in the two groups could reflect better compliance in the experimental group. Moreover, at baseline, the high-flavanol chocolate group had theobromine concentrations that tended to be higher compared with the low-flavanol chocolate group. Therefore, the experimental group could have had a higher consumption of dietary sources of theobromine from their regular diet at baseline and throughout the study, even though all women were instructed to avoid these foods.

Overall, 42 of 44 randomized pregnant women were retained in our study, and only 1 participant was not included in intention-to-treat analysis.

Weight increases linearly during the 2^nd^ and 3^rd^ trimesters of pregnancy, but is associated with escalating physiological weight in pregnancy. Mean gestational weight gain by our pregnant study participants was in accordance with new recommendations of the committee re-examining pregnancy weight guidelines [38]. Caloric intake and nutritional dietary composition did not change significantly during the trial. Although chocolate has high energy density and is perhaps recognized as a fattening food, several studies [35,39-41] have not discerned any weight increase after daily chocolate intake. In our pilot RCT, participants in the 2 arms were instructed not to add chocolate bars to their usual diet but to use them by replacing foods of similar energy and macronutrient composition. Moreover, to ensure support and motivation for adequate use of chocolate, women were met individually by a dietitian who offered global counseling. These strategies could have a favorable impact on weight gain during pregnancy. Therefore, we can speculate that weight gain was not attributable to chocolate intake.

The participants were in good health at baseline and probably represented mainly females with an interest in health and diet. Therefore we can speculate that it may have contributed to reduce the possibility of a significant chocolate intake effect on FMD and BP. Therefore, our results may not be applicable to high-risk pregnant women.

## Conclusion

The results of this pilot RCT confirm the feasibility of a large RCT and flag important methodological, physiological and clinical elements that need to be taken into account in large-scale clinical studies. Treatment compliance by all participants was satisfactory. Consumption of flavanol-rich chocolate was not associated with significant changes in FMD, SBP and DBP in pregnant women with normal BP. We cannot rule out that failure to improve could have been due to a ceiling effect.

We could hypothesize that the absence of effects was attributable to our healthy, normotensive female population, among whom only small changes in BP or FMD were to be expected. Nevertheless, as our study results did not show side-effects, it would be pertinent to specifically investigate these outcomes of flavanol-rich chocolate in pregnant women at high risk of PE.

## Abbreviations

BMI: Body mass index; BP: Blood pressure; DBP: Diastolic blood pressure; FFQ: Food frequency questionnaire; FMD: Flow-mediated dilation; HPLC: High-pressure liquid chromatography; MAP: Mean arterial blood pressure; NO: Nitric oxide; PE: Preeclampsia; RCTs: Randomized clinical trials; SBP: Systolic blood pressure.

## Competing interests

All other authors declare that they have no conflicts of interest.

## Authors’ contributions

JAM analyzed and interpretation the data and wrote the paper. EB performed the experiments, acquisition of data and revising critical. SL acquisition of data and revising critical. CB conception and design, performed the experiments, acquisition of data, analyzed and interpretation the data and revising critical. LB acquisition of data and revising critical. CC acquisition of data and revising critical. MN Performed the experiments, acquisition of data and revising critical. SD Conception and design, analyzed and interpretation the data, wrote the paper and revising critical. All authors read and approved the final manuscript.

## Protocol

Full details of the trial protocol can be requested: sylvie.dodin@oqy.ulaval.ca
